# Assessment of Sourdough Fermentation Impact on the Antioxidant and Anti-Inflammatory Potential of Pearl Millet from Burkina Faso

**DOI:** 10.3390/foods13050704

**Published:** 2024-02-26

**Authors:** Morena Gabriele, Andrea Cavallero, Elena Tomassi, Nafiou Arouna, Július Árvay, Vincenzo Longo, Laura Pucci

**Affiliations:** 1 Institute of Agricultural Biology and Biotechnology, National Research Council, 56124 Pisa, Italy; andrea.cavallero@ibba.cnr.it (A.C.); elena.tomassi@ibba.cnr.it (E.T.); nafiou.arounaa@gmail.com (N.A.); vincenzo.longo@ibba.cnr.it (V.L.); laura.pucci@ibba.cnr.it (L.P.); 2 Department of Agricultural Sciences, University of Naples Federico II, Portici, 80055 Naples, Italy; 3 Institute of Food Sciences, Faculty of Biotechnology and Food Sciences, Slovak University of Agriculture, 949 76 Nitra, Slovakia; julius.arvay@uniag.sk

**Keywords:** pearl millet, sourdough fermentation, antioxidant capacity, HT-29 cell line, anti-inflammatory capacity

## Abstract

Millet, a gluten-free cereal, has received attention for its environmental friendliness and higher protein content than other grains. It represents a staple food in many African countries, where fermentation is traditionally used for preserving food products and preparing different cereal-based products. This study aimed to assess the impact of sourdough fermentation on bioactive compounds and antioxidant and anti-inflammatory properties of pearl millet from Burkina Faso. Phenolic compounds were investigated spectrophotometrically and by HPLC-DAD. The antioxidant activity of unfermented (MF) and fermented (FeMF) millet was evaluated in vitro by spectrophotometric and fluorometric assays and ex vivo on oxidized human erythrocytes for hemolysis inhibition. Finally, the potential anti-inflammatory effect of FeMF and MF was evaluated on human adenocarcinoma cell line (HT-29) exposed to TNF-α inflammatory stimulus. Results revealed significantly higher levels of polyphenols, flavonoids, and in vitro antioxidant activity following millet fermentation. Notable differences in phenolic composition between FeMF and MF are observed, with fermentation facilitating the release of bioactive compounds such as gallic acid, quercetin, and rutin. A dose-dependent protection against oxidative hemolysis was observed in both FeMF- and MF-pretreated erythrocytes. Similarly, pretreatment with FeMF significantly reduced the levels of inflammatory markers in TNF-α-treated cells, with effects comparable to those of MF. Fermentation with sourdough represents a simple and low-cost method to improve the bioactive compounds content and in vitro antioxidant activity of millet flour with promising nutraceutical potential.

## 1. Introduction

Millet, a group of small-seeded gluten-free cereal grains, has long been consumed as a staple food in many regions across the world [1]. Recognizing its environmental friendliness and exceptional resilience to drought and pests, the Food and Agriculture Organization (FAO) designated 2023 as the “International Year of Millet” [2]. Particularly in arid and semi-arid regions grappling with the impacts of climate change, millets’ intrinsic hardiness, especially their minimal water requirements, positions them as vital contributors to resilient agricultural systems [3].

During the last years, the interest in millet and its potential as a nutritious and functional food has increased [4]; millet grains offer a wealth of essential nutrients, dietary fiber, and bioactive compounds such as phenolic acids and flavonoids, which contribute to their healthy properties [1,5,6,7]. Indeed, epidemiological studies have consistently demonstrated a lower incidence of diabetes among individuals who regularly consume millets, suggesting a beneficial role of millets in modulating blood glucose release [1,8,9].

Moreover, millet and, in particular, pearl millet have a higher protein content compared to other cereals like sorghum and wheat [10]. Additionally, the application of fermentation techniques to millet flour has been explored to enhance its nutritional value and functional attributes [6].

Fermentation, a traditional food processing technique, has been utilized to ameliorate the nutritional and functional properties of various food commodities. Especially the fermentation with sourdough, due to its diversified microbial composition, increases the content of bioactive substances, improves protein digestibility, increases the bioavailability of minerals, lowers the glycemic index, allows for the enrichment of dietary fiber, and decreases the content of anti-nutritional factors [11,12]. In particular, the use of natural sourdough has shown a remarkable improvement in the content of bioactive molecules with high antioxidant power in many cereals in our previous articles [13,14].

Fermentation of millet flour has gained attention as a means to enhance its digestibility, bioavailability of nutrients, and release of bioactive compounds [15]. During fermentation, microorganisms, such as yeasts and lactic acid bacteria, metabolize the carbohydrates and proteins in millet flour, resulting in the production of organic acids, vitamins, enzymes, and beneficial metabolites [16,17]. These fermentation-derived products contribute to the improved nutritional quality and functional properties of millet flour [18]. Furthermore, during fermentation, enzymatic hydrolysis processes occur, resulting in the liberation of a vast array of bioactive peptides. These peptides have a diverse range of activities, which include anti-thrombotic, antimicrobial, antihypertensive, immunomodulatory, and antioxidative properties [19]. Cereal-fermented foods have been related to a reduced risk of cardiovascular diseases, diabetes, and cancer due to the presence of antioxidant molecules that can counteract the harmful effects of free radicals [20,21,22]. However, in unfermented cereals, the majority of natural antioxidants, like phenolic compounds, are often bound to cell walls, glycosylated, or in polymeric forms which affects their bio-accessibility [23,24].

Nowadays, the rising attention to a balanced and healthy diet has led to an increased demand for novel functional foods that can mitigate the risk of various pathologies associated with inflammation [25]. While several studies have examined the nutritional and functional changes that occur during millet fermentation, there is a need for an exhaustive understanding of the impact of fermentation on the bioactive compounds, antioxidant, and anti-inflammatory activity of millet flour [24]. Additionally, the potential health benefits of consuming fermented millet flour and its applications in the development of functional foods warrant further investigation.

This work dealt with the impact of fermentation with sourdough on the nutraceutical properties of pearl millet from Burkina Faso. This gluten-free cereal represents a staple food in many African countries, where fermentation is the principal process for preserving food products and is traditionally used to prepare numerous cereal-based products. Therefore, this work aimed to evaluate the effect of sourdough fermentation on the phytochemical content and antioxidant capacity of the pearl millet flour (*Pennisetum glaucum* L.) by in vitro assays and as hemolysis inhibition on oxidized human erythrocytes. Moreover, the anti-inflammatory potential of fermented and unfermented millet flour was evaluated on the human adenocarcinoma cell (HT-29) line under inflammatory stimulus. Through a comprehensive analysis, the work proposes to shed light on the potential benefits and applications of fermented millet flour as a functional ingredient in the food industry.

## 2. Materials and Methods

### 2.1. Chemicals and Reagents

All standards and reagents were of analytical grade. Sodium acetate, sodium carbonate, sodium hydroxide, potassium chloride, potassium persulfate, catechin hydrate, gallic acid, fluorescein sodium salt, quercetin dihydrate, 2,2′-azobis (2-amidinopropane) dihydrochloride (AAPH), 2,2-azinobis(3-ethylbenzthiazoline-6-sulphonic acid) diammonium salt (ABTS), 1,1-diphenyl-2-picrylhydrazyl (DPPH), 2′,7′-dichlorofluorescein diacetate (DCFH-DA), Folin-Ciocalteu reagent, 6-hydroxy-2,5,7,8-tetramethylchromane-2-carboxylic acid (Trolox), dimethyl sulfoxide (DMSO), iso-quercitrin, 4-OH benzoic acid, rutin, trans-ferulic acid, trans-p-coumaric acid, vanillic acid, vitexin, acetonitrile (HPLC grade), ethanol, hydrochloric acid, methanol (HPLC grade), phosphoric acid (ACS grade), trichloroacetic acid, cell culture media and supplements were purchased from Sigma-Aldrich (St. Louis, MO, USA). Aluminum chloride and sodium nitrite were purchased from Carlo Erba (Milan, Italy). A Simplicity 185 purification system (Millipore SAS, Molsheim, France) was used to treat double deionized water (ddH_2_O; 18.2 MΩ/cm, 20 °C).

### 2.2. Plant Material and Extraction

Pearl millet, i.e., *Pennisetum glaucum* L. based on sample caryopses morphological evaluation, was purchased from markets in Burkina Faso. Millet flour was obtained by grinding millet grains using a laboratory mill. The fermentation of millet flour was carried out for 96 h using a natural sourdough consisting of different species of Lactobacilli, mainly *L. sanfranciscensis* and *L. pentosus*, and yeast strains, as previously described by Balli et al. [24]. Water (60%) was added to moisten the millet flour (40%), and then the sourdough, provided by “Lievitamente s.r.l.” (Viareggio-LU, Italy), was added to initiate fermentation. The fermentation temperature was maintained at around 38 °C, while the pH reached a value of 4. Once fermented, the product was frozen and lyophilized for two days using a freeze-dryer Lyovac GT 2 (SRK Systemtechnik, Riedstadt, Germany). The unfermented (MF) and fermented (FeMF) millet flour was suspended with 10% DMSO in distilled water, sonicated (three cycles: 10 s on/10 s off), and shaken gently for 1 h; samples were centrifuged for 10 min at 2300× *g* at 4 °C (Jouan CR3i centrifuge, Newport Pagnell, UK), and the supernatant was collected, filtered (0.2 µm VWR International PBI, Milan, Italy), and kept at 4 °C in the dark until use [26]. The extraction was carried out in triplicate. These extracts were used for all the analyses if not otherwise specified.

### 2.3. Total Polyphenols and Flavonoids Content

The unfermented and fermented millet extracts were screened for total polyphenol and flavonoid content as described by Kim et al. [27], with some modifications. Total polyphenols, estimated as Folin-Ciocalteu (FC) reducing capacity, were expressed as mg of gallic acid equivalents (GAE)/g dry weight (dw). Extracts or standard (100 μL) were mixed with 0.2 N Folin-Ciocalteu reagent (500 μL) and incubated for 5 min in the dark. Then, 0.7 M sodium carbonate (400 μL) was added, and the absorbance was measured at 760 nm, after 2 h of incubation at room temperature in the dark. Total flavonoids were quantified using the aluminum chloride colorimetric method and expressed as mg catechin equivalents (CE)/g dw. Extracts or standard (200 μL) were mixed with dH_2_O (800 μL) and 5% sodium nitrite (60 μL) for 5 min at room temperature. Then, 10% aluminum chloride (60 μL) was added, incubated for 6 min, and finally reactions were neutralized with 1M sodium hydroxide (400 μL). Absorbance was measured at 430 nm after 30 min of incubation at room temperature.

### 2.4. Quantification of Polyphenols by HPLC-DAD

The unfermented and fermented millet samples (3 g) were extracted with 20 mL of 80% methanol at laboratory temperature for 2 h by horizontal shaker Unimax 2010 (Heidolph Instruments, GmbH, Schwabach, Germany). The extract was filtered through Munktell No 390 paper (Munktell & Filtrak GmbH, Bärenstein, Germany) and stored in closed 20 mL PE vial tubes. Before HPLC analysis, the extract was filtered through syringe filter Q-Max (0.22 µm, 25 mm, PVDF) (Frisenette ApS, Knebel, Denmark). All compounds were determined using an Agilent 1260 Infinity HPLC (Agilent Technologies GmbH, Wäldbronn, Germany) with quaternary solvent manager coupled with degasser (G1311B), sampler manager (G1329B), column manager (G1316A) and DAD detector (G1315C). All HPLC analyses were performed on a Purosphere^®^ reverse phase C18 column (250 mm × 4 mm × 5 µm) (Merck KGaA, Darmstadt, Germany). The mobile phase consisted of gradient acetonitrile (A) and 0.1% phosphoric acid in ddH_2_O (B). The gradient elution was as follows: first, 0–1 min isocratic elution (20% A and 80% B), 1–5 min linear gradient elution (25% A and 75% B), 5–15 min (30% A and 70% B) and 15–25 min (40% A and 60% B). The post-run was 3 min. The initial flow rate was 1 mL/min, and the injection volume was 5 μL. Column thermostat was set up to 30 °C and the samples were kept at 4 °C in the sampler manager. The detection wavelengths were set up at 265 nm (4-OH benzoic acid, vanillic acid, rutin), 320 nm (gallic acid, vitexin, trans-*p*-coumaric acid, trans-ferulic acid), and 372 nm (iso-quercitrin, quercetin). Data were collected and processed using Agilent Open Lab Chem Station software 2.8 for LC 3D systems.

### 2.5. In Vitro Antioxidant Activity

#### 2.5.1. DPPH Radical Scavenging Activity

The DPPH assay was carried out for the evaluation of the radical scavenging activity of MF and FeMF extracts. An aliquot of extract (50 μL) was added to a methanolic solution of DPPH 60 μM (1950 μL) and mixed for 30 min in the dark at 30 °C. The absorbance was recorded at 517 nm by a Perkin-Elmer Lambda 365 spectrophotometer and the extract concentration corresponding to 50% DPPH inhibition (EC_50_) was determined according to Guimaraes et al. [28].

#### 2.5.2. ABTS Radical Scavenging Activity

The ABTS assay was carried out according to the method described by Chelucci et al. [29]. ABTS radical cations (ABTS+) were produced through the oxidation of ABTS using potassium persulfate and were subsequently reduced by antioxidants that donate hydrogen. Fresh ABTS stock solution (80 μL of 140 mM potassium persulfate added to 5 mL 7 mM ABTS) was shaken overnight in the dark, then diluted with methanol to obtain an absorbance of 0.7 ± 0.02 at 734 nm (Perkin Elmer UV/VIS Lambda 365, Waltham, MA, USA). Lastly, 190 μL of extract were added to 1 mL of ABTS diluted solution, and the absorbance was read at 734 nm after 10 min incubation. Trolox served as the standard antioxidant. The extent of ABTS+ inhibition was determined, and the result was presented as μmol Trolox equivalent/g dw (TEAC).

#### 2.5.3. Ferric Reducing Antioxidant Power (FRAP) Assay

The FRAP assay was carried out according to Colosimo et al. [14] and 85 µL of aqueous extracts were mixed with 2500 µL of freshly prepared solution of FRAP containing TPTZ 10 mM in HCl 40 mM, FeCl_3_·6H_2_O 20 mM, and acetate buffer 300 mM (pH 3.6) at a ratio of 1:1:10. The sample was gently vortexed and incubated at room temperature for 6 min. The absorbance was then recorded at 593 nm, and the results, expressed as Fe^2+^ equivalent (µM), were estimated using a calibration curve of an aqueous solution of FeSO_4_·7H_2_O (100–2000 µM).

#### 2.5.4. Oxygen Radical Absorbance Capacity (ORAC) Assay

The ORAC assay was carried out as previously described [29]. AAPH was used as a peroxyl radical generator and fluorescein as a probe. The final reaction mixture contained 0.04 mM fluorescein sodium salt in 0.075 M phosphate buffer, pH 7.4, diluted sample, or 5 mM Trolox. The control was 0.075 M phosphate buffer, pH 7.4. The fluorescence decay of fluorescein was measured at λ_ex_ 485 nm and λ_em_ 514 nm (VictorTM X3 Multilabel Plate Reader, Waltham, MA, USA), with Trolox utilized as a standard. ORAC values were quantified in μmol Trolox equivalent (TE)/100 g dw.

### 2.6. Oxidative Hemolysis of Erythrocytes

Human blood samples were centrifuged at 2300× *g* at 4 °C for 10 min. After discarding the buffy coat and plasma, erythrocytes were washed twice with PBS pH 7.4. The hemolysis of erythrocyte was quantified at 540 nm according to Mikstacka et al. [30], by measuring the hemoglobin (Hb) released in the supernatant of human erythrocyte pre-treated with FeMF and MF extracts (0.2, 0.4 and 0.8 mg mL^−1^), then exposed to AAPH peroxyl radicals. Results were presented as a percentage of hemolysis inhibition relative to the control (AAPH-only treated cells). Erythrocytes not treated with AAPH served as a blank sample to account for spontaneous hemolysis.

### 2.7. Human Intestinal Cell Culture

The HT-29 (human colonic adenocarcinoma, DSMZ, Braunschweig, Germany) cell line was cultured in DMEM/F12 medium supplemented with 100 µg mL^−1^ streptomycin, 100 units mL^−1^ penicillin, and 10% fetal bovine serum at 37 °C in a 5% CO_2_ humidified incubator. For treatments, DMEM/F12 medium devoid of phenol red and FBS but containing antibiotics was used. Before exposure to extracts or TNFα, cells underwent a serum starvation phase of 1 h. Following this, HT-29 cells were either pre-treated for 1 h with 0.08 and 0.4 mg mL^−1^ of MF and FeMF extract or not, before being stimulated for 24 h with or without 5 ng mL^−1^ of TNFα. HT-29 were seeded into a 96-well plate (10^4^ cells/well) and cell viability was evaluated by MTT assay as previously described [26].

### 2.8. RT-PCR and Quantitative Real-Time RT-PCR

For gene expression analysis, HT-29 were seeded into a 6-well plate and treated once confluent. Total RNA was extracted and reverse-transcribed using the E.Z.N.A.^®^ Total RNA Kit I (OMEGA bio-tek, Norcross, GA, USA) and the iScriptTM cDNA Synthesis Kit (Bio-Rad, Hercules, CA, USA), respectively. Then, the SsoFastTM EvaGreen^®^ Supermix (Bio-Rad, Hercules, CA, USA) and β-actin, ICAM-1, IL-8, BAX, COX-2, and HO-1 primers [26] were used for quantitative real-time RT-PCR analysis (CFX Connect Real-Time PCR Detection System, Bio-Rad, Hercules, CA, USA). Each gene was analyzed in triplicate, and its expression was determined using the 2^−ΔΔCT^ method for relative quantification [26]. Results are presented as the fold change in expression levels relative to the control samples.

### 2.9. Statistical Analysis

Statistical analysis was carried out using GraphPad Prism 9 for macOS (GraphPad Software, San Diego, CA, USA). The results were expressed as mean values ± standard deviation (SD) after carrying out the experiments in triplicate. ANOVA (One-way analysis of variance) using Dunnett’s multiple comparison tests was used to examine sample differences. For FeMF and MF comparison unpaired Student’s *t*-test was used. A *p*-value lower than 0.05 is considered as statistically significant.

## 3. Results

### 3.1. Content of Bioactive Compounds and Antioxidant Activities In Vitro

The bioactive compound content and the in vitro antioxidant activities, presented in Table 1, were determined in both fermented (FeMF) and unfermented millet flour (MF).

As demonstrated in Table 1, the levels of bioactive compounds exhibited a significant increase following millet fermentation. Specifically, total polyphenols were approximately 1.7-fold, and the flavonoid content was approximately 2.2-fold in the FeMF flour compared to the MF counterpart.

In terms of in vitro antioxidant activity, various assays were employed, including DPPH, FRAP, ABTS, and ORAC. Similarly, fermentation substantially enhanced the antioxidant properties of millet flour. The FeMF sample displayed a slight increase in FRAP (1.1-fold), ABTS (1.2-fold), and ORAC (1.3-fold) values. Moreover, a 1.7-fold reduction of EC_50_ compared to the MF sample was observed for the DPPH assay.

### 3.2. HPLC-DAD Quantification of Phenolic Compounds

Table 2 presents the analysis of phenolic compounds in both MF and FeMF flours utilizing HPLC-DAD. Gallic acid, rutin, and quercetin were exclusively identified in the fermented millet flour. Instead, trans-*p*-coumaric acid was detected only in MF. The presence of 4-OH benzoic acid, vitexin, and vanillic acid was observed in both fractions, but their concentrations were significantly higher in fermented millet, exhibiting a fold increase of 1.4, 1.7, and 5.2, respectively.

### 3.3. Hemolysis Test

**Figure 1:**
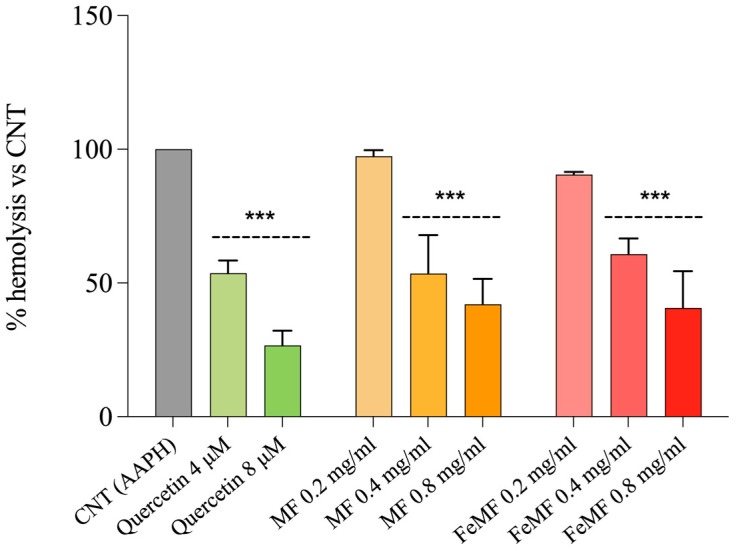
Protective effect of increasing concentrations (0.2, 0.4, and 0.8 mg mL^−1^) of fermented (FeMF) and unfermented (MF) millet extracts on AAPH-induced hemolysis in erythrocytes. Quercetin (4 and 8 µM) was used as a standard. Results are the mean ± SD of at least three replicates. One-way ANOVA followed by Dunnet’s Multiple Comparison test: significantly different from control, AAPH-treated cells (100% hemolysis), *** *p* < 0.001.

The protective effect of growing concentrations of MF and FeMF flour extract toward AAPH-induced hemolysis stress in erythrocytes is illustrated in Figure 1.

As shown in Figure 1, both MF and FeMF, at 0.4 and 0.8 mg mL^−1^, showed lysis protection values superimposable to the highest quercetin concentration (8 µM). Moreover, compared to control cells, MF and FeMF at the same doses significantly protected, with comparable effects, erythrocytes from oxidative hemolysis (*p* < 0.001 vs. control). Therefore, millet fermentation does not improve or worsen the ability of millet to protect erythrocytes from oxidative hemolysis.

### 3.4. Protective Effect against TNFα-Induced Intestinal Alterations of FeMF and MF Extracts

**Figure 2:**
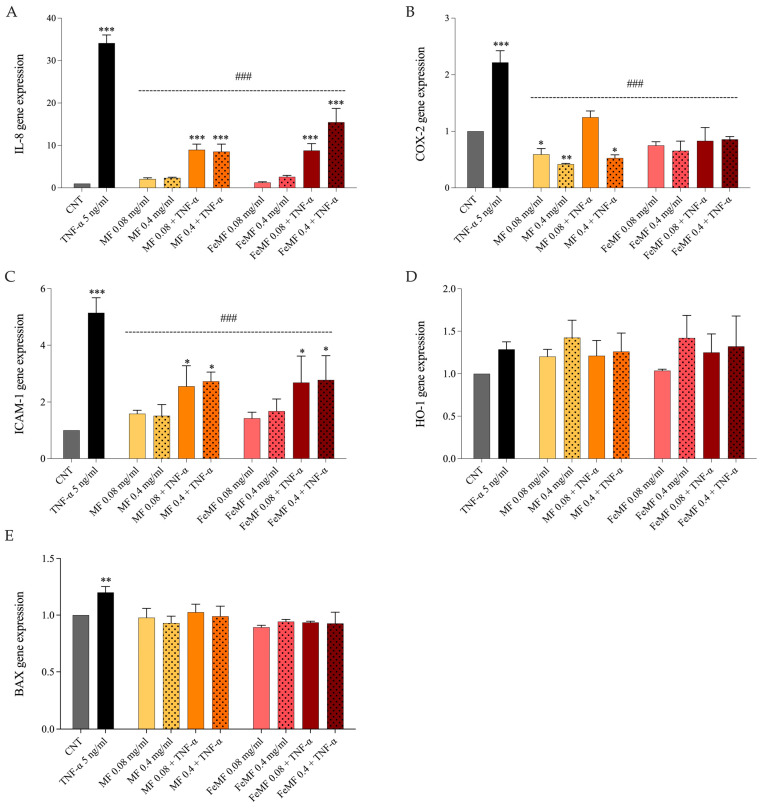
Evaluation of IL-8 (**A**), COX-2 (**B**), ICAM-1 (**C**), HO-1 (**D**), and BAX (**E**) expression by quantitative real-time RT-PCR in HT-29 cells. Cells were pre-treated for 1 h with either 0.08 or 0.4 mg mL^−1^ of fermented (FeMF) or unfermented (MF) millet extract, followed by a 24 h exposure to 5 ng mL^−1^ TNF-α. Experiments were carried out in triplicate and results are presented as the fold-change in expression levels relative to the control samples. One-way ANOVA with Dunnett’s multiple comparison test: * different from control (CNT), * *p* < 0.05, ** *p* < 0.01, *** *p* < 0.001; different from TNF-α, ### *p* < 0.001.

The potential protective effect of MF and FeMF against TNF-α-induced alterations was investigated in a human colonic adenocarcinoma cell line. Cell viability was determined by MTT assay to ensure the absence of cytotoxic effects. Subsequently, the effect of MF and FeMF was evaluated under basal conditions and during TNF-α-induced inflammation by examining the gene expression levels of markers associated with inflammation (IL-8, COX-2, and ICAM-1), oxidative stress (HO-1), and apoptosis (BAX) (Figure 2).

Under the basal condition, pre-treatment with MF and FeMF did not alter the expression of IL-8, ICAM-1, HO-1, and BAX. Instead, pre-treatment with MF, at both concentrations, reduced considerably the basal level of COX-2 (*p* < 0.05 and *p* < 0.01 vs. control).

Conversely, TNF-α treatment led to an upregulation of all genes involved in inflammation (IL-8, COX-2, and ICAM-1; *p* < 0.001 for all) and apoptosis (BAX; *p* < 0.01) compared to the control cells. Under the inflammatory condition, pre-treatment with MF, at both concentrations, effectively mitigated the effects induced by TNF-α exposure. It significantly decreased the expression of IL-8 (*p* < 0.001), COX-2 (*p* < 0.001), and ICAM-1 (*p* < 0.001) while not affecting the expression levels of HO-1 and BAX. Similarly to MF, FeMF significantly reduced IL-8, COX-2, and ICAM-1 gene expression (*p* < 0.001 for all) in TNF-α-induced inflamed cells. Therefore, fermentation of millet with sourdough did not improve or worsen the anti-inflammatory potential of millet flour.

## 4. Discussion

This work investigated the impact of sourdough fermentation on the bioactive composition and content of millet flour, together with its antioxidant and anti-inflammatory properties. Millet is an interesting gluten-free cereal, suitable for individuals with celiac disease, with similar or higher nutritional properties than conventional cereals, thanks to its high energy values, lipids, high-quality proteins, and a source of dietary fiber and micronutrients like calcium, iron, and zinc [31], besides climate-change resilient characteristics and low glycemic index suitable for diabetic patients [32].

Results showed that fermentation with sourdough increased the total polyphenol and flavonoid content of millet flour by approximately 1.7- and 2.2-fold, respectively. Moreover, following fermentation, we observed a change in the phenolic HPLC profile. The increased content of total polyphenols and flavonoids is in line with Gabaza et al. [33]. Moreover, fermentation enhances the release of flavonoids from cereals and induces the transformation of various polyphenolic compounds, improving their antioxidative activity [16]. Notably, the activity of enzymes produced and released by lactic bacteria during fermentation plays a crucial role in breaking down cereal cells, facilitating the extraction of flavonoids, and promoting the depolymerization of high molecular weight phenolic compounds [34,35]. Consequently, fermentation emerges as a valuable method for augmenting the availability of natural antioxidants.

The HPLC-DAD analysis revealed different phenolic compound concentrations and profiles in fermented and unfermented millet flour. The results indicated notable disparities in the phenolic compositions of the two types of flours. Among them, vitexin was the most abundant in fermented millet, followed by 4-OH benzoic acid, gallic acid, and vanillic acid. Moreover, gallic acid, quercetin, and rutin were exclusively identified in the fermented millet flour, indicating that fermentation might facilitate the release of these compounds, as supported by the literature [36]. These bioactive components enhance the body’s redox defense, preventing oxidative stress and cellular injury caused by free radicals [36]. Contrary to the findings of Salar et al. [37], trans-*p*-coumaric acid was detected only in the unfermented millet flour.

The fermented millet flour showed an important increase in the in vitro antioxidant activities, compared to the unfermented one, in line with the increment in the phenolic and flavonoid content. Microbial enzymes, produced by fermentation can hydrolyze glucosides, and break down plant cell walls or starch, facilitating the extraction of bio-compounds, like quercetin and rutin, improving total phenolic content and their free radical scavenging capacity [16,38,39]. No significant increase was observed in the ABTS test values. This assay is based on the single-electron transfer (SET) mechanism, otherwise, the antioxidant tests DPPH, FRAP, and ORAC are based on a mechanism accompanied by the donation of a hydrogen atom (HAT) [40,41]. The latter correlates with the quantity of polyphenols and also with the quality of the phenolic compound, based on the number of hydroxyl groups in the molecule [42].

The hemolysis test was used to evaluate the antioxidant properties of bioactive compounds relying on the inhibition of lipid peroxidation, induced by AAPH radicals, on the erythrocyte membrane. The ability of the antioxidant to scavenge the peroxyl radicals correlates with the antihemolytic activity [43]. Our results indicate that both FeMF and MF, at the highest concentrations, provide significant protection against oxidative hemolysis compared to control cells, exposed only to AAPH, which show the highest hemolysis values. The level of protection provided by both MF and FeMF is comparable to the highest dose of quercetin, a well-known antioxidant compound [44]. These findings demonstrate that both MF and FeMF possess good anti-hemolytic properties, indicating their potential as protective agents against oxidative damage in erythrocytes; moreover, based on our results, the fermentation process did not improve or worse the antioxidant potential of millet flour against erythrocytes lysis.

Finally, the anti-inflammatory effect of pre-treatment with FeMF and MF extracts on the HT-29 inflamed cell model was investigated. The cells were exposed to TNF-α as an inflammatory stimulus for 24 h, and the expression of genes involved in inflammation (IL-8, COX-2, and ICAM-1), oxidative stress (HO-1), and apoptosis (BAX) was assessed. The results showed that pre-treatment with MF, at both concentrations (0.08 and 0.4 mg mL^−1^), was effective in mitigating the alterations induced by TNF-α exposure. This is evidenced by a significant decrease in the expression levels of IL-8, COX-2, and ICAM-1 genes, indicating the potential anti-inflammatory effects of MF pre-treatment. These findings are consistent with previous observations in lipopolysaccharide-inflamed HT-29 cells pre-treated with bound polyphenols from foxtail millet bran [45]. Instead, the expression levels of HO-1 and BAX genes were not affected by MF pre-treatment. Contrary to what was observed with fermented *T. dicoccum* (spelt) flour [13], our results indicate that the fermentation of millet flour with sourdough did not improve the anti-inflammatory properties of unfermented flour. Indeed, both MF and FeMF showed similar abilities in reducing IL-8, COX-2, and ICAM-1 gene expression in TNF-α-induced inflamed cells, although higher phenolic compounds were detected in FeMF than MF.

## 5. Conclusions

Our study points out that sourdough fermentation improves the phenolic profile and in vitro antioxidant capacity of millet flour while not affecting its anti-hemolytic and anti-inflammatory properties. Both fermented and unfermented millet flour provided significant dose-dependent protection to human erythrocyte cells against oxidative hemolysis. Similarly, pretreatment with fermented millet significantly reduced IL-8, ICAM-1, and COX-2 gene expression in inflamed HT-29, with comparable effects to those obtained with unfermented flour. The simplicity and affordability of the fermentation make it a practical choice for enhancing the overall quality of millet; in future studies, the selection of a single strand of lactic acid bacteria or a consortium of selected yeast and lactobacillus could be effective in improving the antioxidant and anti-inflammatory properties of millet on human cells, conferring an additional nutraceutical and nutritional value to millet flour.

## Figures and Tables

**Table 1:** Comparison of total polyphenols and flavonoids and in vitro antioxidant activities (DPPH, FRAP, and ORAC) between fermented (FeMF) and unfermented (MF) millet flour. Data represent mean values ± SD from three replicates. Unpaired Student *t*-test: significantly different from MF: * *p* < 0.05, ** *p* < 0.01, *** *p* < 0.001, ^ns^: not statistically significant.

	TP (mg GAE/g dw)	TF (mg CE/g dw)	DPPH EC_50_ (mg mL^−1^)	FRAP (µM Fe^2+^)	ABTS (μmol TE/g dw)	ORAC (μmol TE/100g dw)
FeMF	3.25 ± 0.09 ***	2.84 ± 0.39 *	1.83 ± 0.23 ***	1476.3 ± 0.92 **	19.09 ± 2.64 ^ns^	640.4 ± 30.97 *
MF	1.83 ± 0.02	1.29 ± 0.53	3.16 ± 0.08	1336.2 ± 30.33	15.65 ± 1.95	498.4 ± 78.48

TP: total polyphenols; GAE: gallic acid equivalents; dw: dry weight; TF: total flavonoids; CE: catechin equivalents; DPPH: 2,2-diphenyl-1-picrylhydrazyl; EC_50_: half maximal effective concentration; FRAP: Ferric Reducing Antioxidant Power; TE: Trolox equivalent; ORAC: Oxygen Radical Absorbance Capacity.

**Table 2:** Phenolic compound, concentration (mg/kg dw), wavelengths (nm), class and retention time (Rt, min), in the fermented (FeMF) and unfermented (MF) millet flour. Unpaired Student *t*-test: significantly different from MF: *** *p* < 0.001.

Phenolic Compound	Concentration		Wavelength	Class	Rt
	FeMF	MF			
Gallic acid	106.26 ± 1.30	<LOD	320	Phenolic acid	2.669
4-OH Benzoic acid	141.15 ± 1.24 ***	98.80 ± 3.27	265	Carboxylic acid	4.830
Vanillic acid	39.58 ± 0.55 ***	23.87 ± 0.74	265	Phenolic acid	5.260
Rutin	4.49 ± 0.11	<LOD	265	Flavonol	5.955
Vitexin	296.88 ± 2.50 ***	56.88 ±1.80	320	Flavone	6.275
Iso-Quercitrin	<LOD	<LOD	372	Flavonol	6.987
trans-*p*-Coumaric acid	<LOD	6.48 ± 0.42	320	Hydroxycinnamic acid	7.444
trans-Ferulic acid	<LOD	<LOD	320	Hydroxycinnamic acid	8.219
Quercetin	0.96 ± 0.06	<LOD	372	Flavonol	17.363

LOD: limit of detection.

## Data Availability

The original contributions presented in the study are included in the article, further inquiries can be directed to the corresponding author.

## Abbreviations

AAPH: 2,2′-azobis (2-amidinopropane) dihydrochloride; ABTS: 2,2-azinobis(3-ethylbenzthiazoline-6-sulphonic acid) diammonium salt; BAX: BCL2 associated X, apoptosis regulator; CE: catechin equivalents; COX-2: Cyclooxygenase-2; DMSO: dimethyl sulfoxide; DPPH: 1,1-diphenyl-2-picrylhydrazyl; DPPH: 2,2-diphenyl-1-picrylhydrazyl; dw: dry weight; EC_50_: half maximal effective concentration; FBS: fetal bovine serum; FeMF: fermented millet flour; FRAP: Ferric Reducing Antioxidant Power; FRAP: Ferric Reducing Antioxidant Power; GAE: gallic acid equivalents; HO-1: heme oxygenase-1; HPLC-DAD: High-performance liquid chromatography with diode-array detection; ICAM-1: intracellular adhesion molecule; ICAM-1: Intracellular adhesion molecule-1; IL-8: C-X-C motif chemokine ligand 8; MF: unfermented millet flour; ORAC: Oxygen Radical Absorbance Capacity; TE: Trolox equivalent; TF: total flavonoids; TNF-α: tumor necrosis factor-alpha; TP: total polyphenols; Trolox: 6-hydroxy-2,5,7,8-tetramethylchromane-2-carboxylic acid.
